# Functional identification of NR2 subunits contributing to NMDA receptors on substance P receptor-expressing dorsal horn neurons

**DOI:** 10.1186/1744-8069-4-44

**Published:** 2008-10-10

**Authors:** Chi-Kun Tong, Edward J Kaftan, Amy B MacDermott

**Affiliations:** 1 Department of Physiology and Cellular Biophysics, Columbia University, New York, New York 10032, USA; 2 Department of Neuroscience, Columbia University, New York, New York 10032, USA; 3 Pain Research, Discovery Neuroscience, Wyeth Research, Princeton, NJ 08534, USA

## Abstract

NMDA receptors are important elements in pain signaling in the spinal cord dorsal horn. They are heterotetramers typically composed of two NR1 and two of four NR2 subunits: NR2A-2D. Mice lacking specific NR2 subunits show deficits in pain transmission yet subunit location in the spinal cord remains unclear. We have combined electrophysiological and pharmacological approaches to investigate the composition of functional NMDA receptors expressed by lamina I, substance P receptor-expressing (NK1R+) neurons, as well as NK1R- neurons. Under low Mg^2+ ^conditions (100 μM), the conductance of NMDA receptors at -90 mV (g(-90 mV)) with NR2A or NR2B subunits (NR2A/B) is low compared to conductance measured at the membrane potential where the inward current is maximal or maximal inward current (MIC) (ratio of ~0.07 calculated from Kuner and Schoepfer, 1996). For NR2C or NR2D subunits (NR2C/D), the ratio is higher (ratio ~0.4). NK1R+ and NK1R- neurons express NMDA receptors that give ratios ~0.28 and 0.16, respectively, suggesting both types of subunits are present in both populations of neurons, with NK1R+ neurons expressing a higher percentage of NR2C/D type NMDA receptors. This was confirmed using EAB318, an NR2A/B preferring antagonist, and UBP141, a mildly selective NR2C/D antagonist to increase and decrease the g(-90 mV)/g(MIC) ratios in both subpopulations of neurons.

## Background

NMDA receptors in the spinal cord dorsal horn are key elements in the initiation of changes in synaptic strength [1] and pain hypersensitivity [2,3]. These receptors consist of two obligatory NR1 subunits and two NR2 subunits, of which there are four types encoded by distinct genes: NR2A, NR2B, NR2C and NR2D [4]. The incorporation of different NR2 subunits has a major impact on the functional properties of the NMDA receptor, critically influencing agonist and antagonist affinity, receptor deactivation kinetics, channel conductance and interactions with intracellular proteins [3]. Additionally, NMDA receptors with NR2A or NR2B show higher Mg^2+ ^sensitivity at negative membrane potentials than those with NR2C or NR2D [5,6].

Involvement of NMDA receptors in dorsal horn function has been demonstrated through experiments interfering with expression of different NMDA receptor subunits. Knockdown of the NR1 subunit of NMDA receptors to eliminate functional NMDA receptors in the spinal cord reduces hyperalgesia and allodynia in a number of animal models but does not alter acute pain responses [7-9]. NR2A knockout mice show some reduced forms of hypersensitivities [10-12]. However, these NR2A knockouts display normal acute pain responses [12], formalin-induced hyperalgesia [13] and nerve ligation or injury-induced allodynia [14,15]. NR2B knockout mice do not survive postnatally [16,17], therefore NR2B specific antagonists have been used to study the role of this protein in pain hypersensitivity. Intrathecal administration of NR2B antagonists blocks or decreases PGE2 or NMDA induced allodynia [11] as well as capsaicin-induced hyperalgesia [18]. NR2D knockout mice fail to develop nerve ligation [12], PGE2 [19] or PGF2alpha-induced allodynia [11,20]. Overall, these data suggest that different NR2 subunits are involved in dorsal horn circuits important for the development of hyperalgesia or allodynia but their specific functions remain unresolved.

Lamina I of the spinal cord is a critical site for nociceptive processing, receiving abundant monosynaptic input from nociceptors. The main output neurons of lamina I, the substance P receptor-expressing (NK1R+) projection neurons, are essential in mediating pain hypersensitivity [21,22]. NK1R+ neurons express NMDA receptors [23,24] but little is known about the subtypes of NMDA receptors they express.

In this paper, we have taken advantage of the different magnesium sensitivities and pharmacology of NMDA receptors with different NR2 subunit composition to identify functionally expressed NMDA receptors on NK1R+ and NK1R- dorsal horn neurons in lamina I.

## Methods

### Transverse slice preparation

Lumbar spinal cords were obtained from rats of postnatal day 14 (P14) to P19. The animals were first anesthetized with isoflurane and then decapitated. All experiments were conducted with the approval of the Columbia University Institutional Animal Care and Use Committee and in accord with the Guide for the Care and Use of Laboratory Animals. The spinal cords were excised and placed in ice-cold oxygenated high Mg^2+ ^Krebs solution (95% O_2_/5% CO_2 _saturated Krebs solution, in mM: NaCl 125 or sucrose 250, KCl 2.5, NaHCO_3 _26, NaH_2_PO_4 _1.25, glucose 25, MgCl_2 _6, CaCl_2 _1.5, pH 7.4) plus 1 mM kynurenic acid. After removal of the dura mater and arachnoid membranes, all ventral roots were cut close to the cord and the spinal cord embedded in low melting agarose (Invitrogen Life Technologies) for slicing. Transverse slices (350–450 μm) with attached dorsal roots were obtained using a Leica VT1000S vibrating blade microtome. Slices were then incubated in oxygenated high Mg^2+^Krebs solution (no sucrose included) at 36°C for 1 hour before recording.

### Recording from pre-identified NK1R+ and NK1R- lamina I neurons

The labeling of NK1R+ dorsal horn neurons with fluorescent dye has been described elsewhere [25,26]. In brief, spinal cord slices were incubated in high Mg^2+ ^Krebs solution containing 20 – 40 nM tetramethylrhodamine-conjugated substance P (TMR-substance P) for 20 – 30 minutes at room temperature following 1 hour of recovery at 36°C. After unbound substance P was washed away for at least 20 minutes in an incubation chamber containing oxygenated high Mg^2+ ^Krebs solution, slices were transferred to a submersion style chamber for recording. NK1R+ neurons were identified as expressing NK1R by clear, intense labeling with TMR-substance P. NK1R- neurons were chosen as lamina I neurons showing no evidence of TMR-substance P staining.

### Recording solutions

Intracellular solution used for most of these experiments had the following composition (in mM): Cs-methylsulfonate 130, Na-methylsulfonate 10, EGTA 10, CaCl_2 _1, HEPES 10, QX-314·Cl 5, Mg^2+^-ATP 2, pH adjusted to 7.2 with CsOH, osmolarity adjusted to 290 with sucrose. For some experiments in which intracellular Ca^2+ ^needed to be strongly chelated, BAPTA intracellular solution was used. It was composed of (in mM): Cs-methylsulfonate 50, Na-methylsulfonate 10, BAPTA·Cs 40, CaCl_2 _4, HEPES 10, QX-314·Cl or QX-222·Cl 5, Mg^2+^-ATP 2, TEA·Cl 10, pH adjusted to 7.2 with CsOH, osmolarity about 310. Junction potentials were measured empirically and corrected in the bath before GOhm seal formation for each cell.

Modified Krebs solutions were used for the extracellular bath. To prevent possible neurotoxicity associated with Ca^2+ ^influx through activated NMDA receptors, we replaced 95–98% of the extracellular Ca^2+ ^with 3 mM Ba^2+^. The barium Krebs comprised: NaCl 125, KCl 2.5, NaH_2_PO_4_1.25, NaHCO_3 _26, glucose 25, MgCl_2 _0.1, CaCl_2 _0.04–0.1, BaCl_2 _3 and pH 7.4. TTX (0.5 μM), SR95531 (5–10 μM) and strychnine (1 μM) were included in the extracellular solutions to eliminate action potential generation and involvement of inhibitory circuits.

### Analysis of NMDA induced membrane currents

To obtain the current-voltage relationships of NMDA receptor-mediated currents, NMDA (15 μM) was superfused onto pre-identified, lamina I neurons for 2–3 minutes following several minutes of baseline, whole-cell recording. Triangle voltage ramp commands (the ramp up and ramp down were 0.9 sec duration each) were applied continuously at low frequency (0.05 Hz). Digital sampling frequency was 10 KHz. NMDA applications were repeated 2–3 times before NMDA co-application with antagonists. The data for the first NMDA application were not included for analysis due to changing baseline conditions. Current responses to triangle voltage ramps before and after recovery from NMDA application were averaged as a control current then subtracted from each triangle ramp made during NMDA induced currents. The resulting NMDA current ramps were plotted as a function of membrane potential and further analyzed. To minimize noise for measuring the following parameters, NMDA current ramps were subjected to a rolling average procedure over a 100 msec time frame.

**Figure 1:**
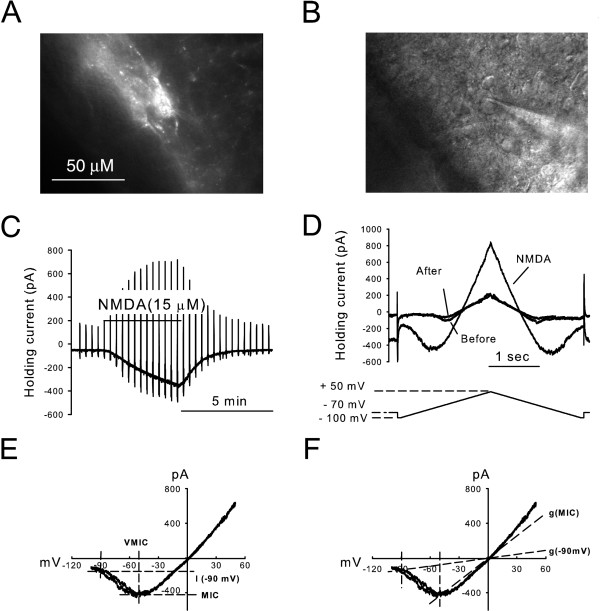
**Parameters representing the voltage sensitivity of current flow through NMDA receptors were determined following NMDA application onto pre-identified dorsal horn neurons.** (**A**) NK1R+ neuron in the superficial dorsal horn was selectively labeled by TMR-substance P in a transverse slice (40 × objective). **(B) **IR-DIC image showing the NK1R+ neuron patched with a pipette. (**C**) A representative trace shows an NMDA-evoked inward current with 100 μM Mg^2+ ^in the bath. (**D**) Current responses to voltage ramps are shown at expanded time base. (**E**) The voltage dependence of NMDA receptor-mediated currents derived from NMDA evoked currents. (**F**) The same as in Figure E. The conductance of NMDA receptors at -90 mV (g(-90 mV)) and MIC (g(MIC)) are illustrated.

For each voltage ramp during NMDA applications, the membrane current at -90 mV (I(-90 mV)), the maximal inward current (MIC), and the membrane potential for the MIC (VMIC) were measured (Figure 1E). The current measured at -90 mV holding potential was determined as I(-90 mV). The MIC was initially determined as the most negative current value in the rolling average. The VMIC was then determined as the voltage corresponding to the MIC. Because each ramp had an up and a down phase, each parameter from a ramp current had a pair of values and they were averaged for following analysis. The conductance at -90 mV and MIC (g(-90 mV) and g(MIC) respectively) as well as conductance ratio were then calculated based on the formulae:

g(-90 mV) = -I(-90 mV)/90 mV

g(MIC)= -MIC/VMIC

g(-90 mV)/g(MIC) = I(-90 mV)*VMIC/(90*MIC)

To compare NMDA receptor g(-90 mV)/g(MIC) ratios under different pharmacological conditions, we averaged three ratio values calculated for each NMDA application near the peak NMDA response at -70 mV. The ratios under different pharmacological conditions or represented by different neuron populations were then compared.

Only cells starting with reversible NMDA induced membrane currents, in which the difference between g(-90 mV)/g(MIC) ratios during wash-in and wash-out of NMDA was less than 0.15, were included for analysis. Cells with high membrane holding current (> -100 pA) were discarded.

### Materials

SR 95531 hydrobromide and QX-222·Cl were purchased from Tocris Cookson (Bristol, UK). QX-314·Cl was purchased from Sigma-Aldrich or Alomone labs (Jerusalem, Israel). Low melting point agarose and TMR-substance P were purchased from Invitrogen Corp. Some TMR-substance P was synthesized and purchased from AnaSpec, Inc. Strychnine was obtained from Sigma-Aldrich. EAB318 was provided by Wyeth Research. EAB318 has IC50 of 20, 80 and 3500 nM for NMDA receptors with NR2A, NR2B and NR2C respectively [27]. UBP141 was synthesized as described [28]. The K_i _of UBP141 for NMDA receptors with NR2A – NR2D are 14, 19, 4 and 2.7 μM respectively [28].

## Results

### The I-V relationship of NMDA currents induced by superfusion of NMDA onto dorsal horn neurons

We investigated the total population of functional NMDA receptors expressed by different classes of lamina I neurons. NMDA was bath-applied onto spinal cord slices to activate all functional NMDA receptors. To identify the type of NMDA receptors expressed by pre-identified lamina I neurons in the spinal cord dorsal horn, we took advantage of the differential sensitivity to Mg^2+ ^inherent in NMDA receptors composed of different NR2 subunits. NMDA receptors containing NR2A or NR2B subunits show more negative slope conductance at negative membrane potentials than those containing NR2C or NR2D [6]. Because the measurable difference in Mg^2+ ^sensitivity is enhanced when extracellular Mg^2+ ^concentration is low, 100 μM extracellular Mg^2+ ^was used throughout these experiments. SR95532 (10 μM), strychnine (1 μM) and TTX (0.5–1 μ;M) were always included to eliminate the inhibitory currents and action potential triggered responses. Most of the Ca^2+ ^in the Krebs was replaced with Ba^2+ ^to diminish evoked neurotransmitter release and Ca^2+ ^dependent currents in the cells.

NK1R+ and NK1R- dorsal horn neurons were visually identified for whole-cell recording following incubation of spinal cord slices in TMR-substance P [25,26] as shown in Figure 1A and 1B, in which fluorescence and IR-DIC images are shown respectively. Bath application of NMDA (15 μM) to these neurons generated inward currents at a holding potential of -70 mV as shown in Figure 1C. To determine the voltage-dependent Mg^2+ ^sensitivity of these receptors, triangle voltage ramp commands from -100 mV to + 50 mV and back to -100 mV were applied before, during and after NMDA application at 0.05 Hz (Figure 1C and 1D). After subtraction of control current, the resulting ramps of NMDA receptor-mediated current were plotted as a function of command voltage as shown in Figure 1E. The voltage sensitivity of the NMDA current generated by the ascending ramp command is similar to that generated by the descending command (Figure 1E). In addition, the current-voltage relationships of these NMDA induced currents had an average reversal potential of -2.0 ± 1.0 mV (n = 15), close to the predicted NMDA receptor reversal potential. The pair of NMDA current responses obtained from each triangle voltage command were used to determine the current at -90 mV (I(-90 mV)), the maximal inward current (MIC) and the membrane potential at which MIC occurs (VMIC) as illustrated in Figure 1E (see Methods). The membrane conductance at -90 mV (g(-90 mV)) and MIC (g(MIC)) were then calculated as shown in Figure 1F.

The voltage sensitivity of NMDA currents depends predominantly on the voltage dependence of Mg^2+ ^block of the receptors [29,30]. The voltage sensitivity of the agonist activated NMDA receptors in our experiments was quantified by dividing g(-90 mV) with g(MIC). The ratio was then compared to the value derived from heterologous expression data using specific NR1 and NR2 subunit combinations. From such data we calculated that NMDA receptors containing NR1/NR2A or NR1/NR2B show g(-90 mV)/g(MIC) ratios of about 0.07 and that their VMIC is between -37 and -40 mV. Conversely, NMDA receptors containing NR1/NR2C or NR1/NR2D have ratios around 0.39 and VMICs around -52 to -57 mV (extracted from Kuner et al. [6]). Thus, for example, lower g(-90 mV)/g(MIC) ratios near 0.07 and less negative VMICs indicate expression of NR2A/B-containing NMDA receptors with high Mg^2+ ^sensitivity.

**Figure 2:**
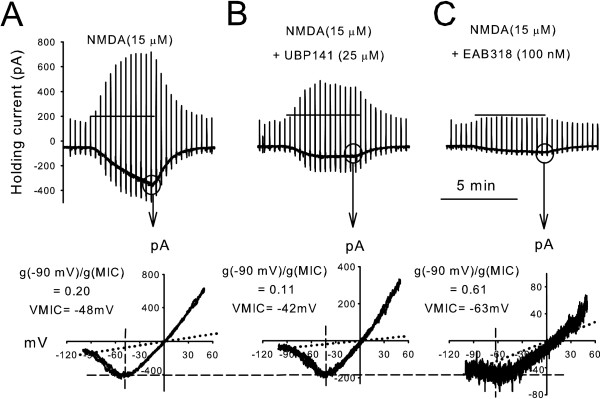
**The voltage-dependence of NMDA receptor-mediated currents is a good indicator of NMDA receptor subtype expression.A-C **show current responses to NMDA bath applied to the same NK1R+ neuron as in Figure 1. (**A**) NMDA-evoked current response is the same as in Figure 1. (**B**) Co-application of NMDA and UBP141, an NR2C/D preferring antagonist, induced a smaller inward current. The I-V relationship shows a more pronounced negative slope conductance. (**C**) Co-application of NMDA and EAB318, an NR2A/B preferring antagonist, induced NMDA receptor-mediated current with less negative slope conductance.

In Figure 2A, the control response of an NK1R+ neuron to 15 μM NMDA is shown. The ramp response recorded when the agonist response to NMDA was at its greatest is plotted at the bottom. The value of the g(-90 mV)/g(MIC) ratio is 0.2. This is intermediate between the values for receptors that include NR2A/B and NR2C/D subunits suggesting that NMDA receptors with both types of NR2 subunits are present on this NK1R+ lamina I neuron. The VMIC value (-48 mV in this example) is also intermediate between the VMIC values of NR2A/B and NR2C/D subunits, supporting the interpretation that the NMDA receptors expressed by this NK1R+ neuron are heterogeneous in NR2 subtype expression.

Although most of the extracellular Ca^2+ ^was replaced with Ba^2+ ^in these experiments, it was still possible that the remaining bath Ca^2+ ^or Ca^2+ ^released from endoplasmic reticulum (ER) was sufficient to trigger activation of other currents, altering g(-90 mV)/g(MIC) ratio and VMIC values. In 9 of 15 cells recorded, intracellular solution containing 40 mM BAPTA was used to fully suppress accumulation of intracellular Ca^2+ ^associated with NMDA receptor activation. There was no significant difference between the g(-90 mV)/g(MIC) values when recording with BAPTA or EGTA intracellular solutions. Thus the data from these two groups were pooled.

### Pharmacological test of NR2 subunit confirms the expression of NR2A/B and NR2C/D type NMDA receptors

Next we used pharmacology to confirm that g(-90 mV)/g(MIC) ratio and VMIC are good indicators of NMDA receptor subtypes expressed by dorsal horn neurons. EAB318 (100 – 200 nM) and UBP141 (20–30 μM) block different NMDA receptor subtypes: UBP141 [28] is a mildly selective NR2C/D preferring antagonist while EAB318 is a NR2A/B selective blocker [27]. If both categories of NR2 subtypes are present, EAB318 should make the NMDA evoked current more NR2C/D like and UBP141 should make the current more NR2A/B like. As predicted, when NMDA was co-applied with UBP141, the current-voltage relationship had a smaller g(-90 mV)/g(MIC) ratio and less negative VMIC (Figure 2A and 2B). This suggests that in the presence of UBP141, a higher proportion of NR2A/B type NMDA receptors dominate the current. When NMDA was co-applied with EAB318, the current voltage relationship shifted to a larger g(-90 mV)/g(MIC) ratio and a more negative VMIC, suggesting that a higher proportion of NR2C/D type NMDA receptors were revealed (Figure 2C).

**Figure 3:**
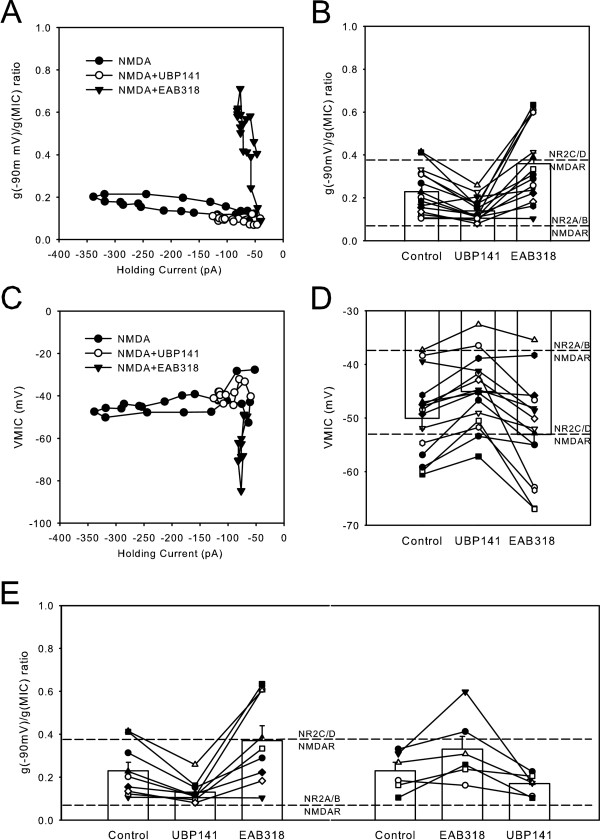
**The NMDA g(-90 mV)/g(MIC) ratio is not a function of the amplitude of NMDA-evoked membrane current. **(**A**) The g(-90 mV)/g(MIC) ratio was calculated for each test ramp throughout NMDA applications in the absence and presence of antagonists and plotted as a function of NMDA-evoked current amplitude. (**B**) Summary of the antagonist effects on the g(-90 mV)/g(MIC) ratio. (**C**) The VMIC was calculated for each test ramp throughout NMDA applications in the absence and presence of antagonists and plotted as a function of NMDA-evoked current amplitude for the same data as (**A**). (**D**) Summary of the antagonist effects on the VMIC (n = 15). (**E**) The same data as in (B) but grouped according to the sequences of drug application.

To ensure that the shift of g(-90 mV)/g(MIC) ratio and VMIC were genuinely associated with selective block of a subpopulation of NMDA receptors and not simply caused by errors associated with measuring smaller amplitude NMDA evoked currents, the relationship between g(-90 mV)/g(MIC) and NMDA evoked current amplitude was plotted as in Figure 3A. As the NMDA plus antagonists washed onto the dorsal horn neuron under study, the impact of the two antagonists on g(-90 mV)/g(MIC) were different. UBP141 depressed the amplitude of NMDA evoked currents and g(-90 mV)/g(MIC) values. EAB318 also depressed the amplitude of NMDA evoked current but caused a large shift to higher g(-90 mV)/g(MIC) values. In the two situations, UBP141 and EAB318 depressed the peak amplitudes of NMDA induced currents from -306 ± 48 to -130 ± 222 pA (n = 15, p < 0.01 for paired t-test) and -108 ± 16 pA (n = 15, p < 0.01 for paired t-test) respectively. Figure 3B shows the individual and mean g(-90 mV)/g(MIC) ratios determined when NMDA evoked currents were maximal under different drug conditions. UBP141 significantly decreased the g(-90 mV)/g(MIC) ratio from 0.23 ± 0.03 to 0.15 ± 0.01 (n = 15, p < 0.01 for paired t-test) while EAB318 significantly increased the ratio from 0.23 ± 0.03 to 0.36 ± 0.05 (n = 15, p < 0.01 for paired t-test) (see Methods). The upper and lower broken horizontal lines represent the g(-90 mV)/g(MIC) for pure NR2A/B and NR2C/D-containing NMDA receptors respectively as calculated using the data of Kuner and Schoepfer [6]. Comparing our data to these benchmarks shows that the two antagonists are pushing the g(-90 mV)/g(MIC) values in the directions predicted from heterologous expression data, assuming both types of subunits are present in the neuron tested.

We also analyzed VMICs under different drug conditions. Figure 3C, calculated from the same data as Figure 3A, shows the action of the two antagonists on VMIC. As expected, UBP141 caused the VMIC to become somewhat more positive or more NR2A/B like, while EAB318 caused the VMIC to become more negative or more NR2C/D like. Figure 3D is the summary showing that UBP141 significantly shifted the mean VMIC, measured when the currents evoked by NMDA were maximal (see Methods), from -50 ± 2 mV to -45 ± 2 mV (n = 15, p < 0.01 for paired t-test) while EAB318 significantly changed the mean VMIC to -53 ± 2 mV (n = 15, p < 0.05 for paired t-test).

To rule out the possibility that the sequence of antagonist co-application with NMDA may have some effects on the g(-90 mV)/g(MIC) ratio, we grouped the experiments according to the order of drug application. In 9 of 15 cells tested, UBP141 was co-applied with NMDA before EAB318 co-application with NMDA. In 6 of 15 cells tested, UBP141 was co-applied after EAB318. UBP141 significantly decreased the g(-90 mV)/g(MIC) from 0.23 ± 0.04 to 0.13 ± 0.02 (n = 9, p < 0.01 for paired t-test) and EAB318 significantly increased the g(-90 mV)/g(MIC) from 0.23 ± 0.04 to 0.37 ± 0.07 (n = 9, p < 0.01 for paired t-test) when UBP141 was applied first (Figure 3E left side). Similarly, EAB318 significantly increased the g(-90 mV)/g(MIC) from 0.23 ± 0.04 to 0.33 ± 0.06 (n = 6, p < 0.05 for paired t-test) and UBP141 significantly decreased the g(-90 mV)/g(MIC) from 0.23 ± 0.04 to 0.17 ± 0.02 (n = 6, p < 0.05 for paired t-test) when EAB318 was applied earlier (Figure 3E right side).

### Comparison of NMDA receptor types between NK1R+ and NK1R- neurons

**Figure 4:**
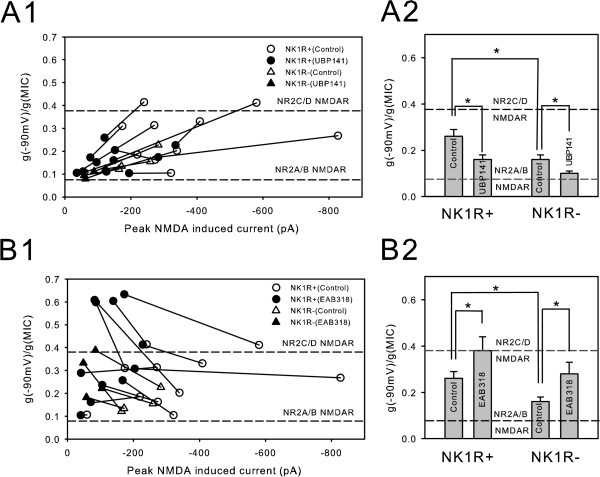
**NK1R+ neurons express higher proportion of NMDA receptors with NR2C/D subunits than do NK1R- neurons.** (**A1**) Individual NK1R+ and NK1R- neurons showed different degree of g(-90 mV)/g(MIC) ratio decrease following exposure to UBP141. (**A2**) Overall, NK1R+ neurons show significantly higher g(-90 mV)/g(MIC) ratio than NK1R- neurons (p < 0.05). (**B1**) Individual NK1R+ and NK1R- neuron showed increased g(-90 mV)/g(MIC) ratio when NMDA was applied in the presence of EAB318. (**B2**) In summary, EAB318 significantly increased g(-90 mV)/g(MIC) ratios in both NK1R+ and NK1R- lamina I neurons.

Having verified that these two approaches, Mg^2+ ^sensitivity and pharmacological blockade, allowed us to distinguish different NMDA receptor subtypes, we asked if NK1+ and NK1- neurons differed in the proportions of NR2A/B and NR2C/D containing receptors that they expressed. Indeed, although co-application of NMDA with either UBP141 or EAB318 significantly changed the g(-90 mV)/g(MIC) ratios, the ratio changes were not the same for all neurons tested. Figure 4A1 shows the UBP141-induced change in the g(-90 mV)/g(MIC) ratio in individual lamina I neurons pre-identified as either NK1R+ or NK1R- neurons. The g(-90 mV)/g(MIC) ratios of most neurons showed high sensitivity to UBP141, suggesting that most dorsal horn neurons express some NMDA receptors that include NR2C/D subunits. On average, NK1R+ neurons had a significantly higher g(-90 mV)/g(MIC) ratio than NK1R- neurons (0.26 ± 0.03, n = 11 v.s. 0.16 ± 0.02, n = 4, p < 0.05 for unpaired t-test), indicating that in these neurons, a higher percentage of NMDA receptors include NR2C/D subunits than in NK1R- neurons (Figure 4A2).

To confirm this observation, we also observed the effect of EAB318 on the g(-90 mV)/g(MIC) ratio of individual neurons in Figure 4B1. EAB318 increased the g(-90 mV)/g(MIC) ratio in most, but not all dorsal horn neurons tested, again suggesting that most of them expressed NMDA receptors with both high and low Mg^2+ ^sensitivity, consistent with the data of UBP141. On average, EAB318 caused the g(-90 mV)/g(MIC) ratio measured from NK1R+ neurons to become more NR2C/D like than from NK1R- neurons (Figure 4B2), consistent with the interpretation that NK1R+ neurons express a higher percentage of NMDA receptors with NR2C/D subunits.

## Discussion

We have identified NMDA receptor subtypes expressed by two populations of dorsal horn neurons; NK1R+ and NK1R- lamina I neurons. Based on our experiments, both highly Mg^2+ ^sensitive (NR2A/B) and poorly Mg^2+ ^sensitive (NR2C/D) NMDA receptors are expressed by NK1R+ neurons. NR2C/D subunits are less strongly expressed by NK1R- lamina I neurons and therefore the NR2A/B receptor subtypes dominate more strongly there.

### Ratio assay confirmed by pharmacology

The main approach for identification of NR2 subunit expression by different neurons in our study was to establish and apply a ratio assay for NMDA receptor-mediated currents recorded in 100 μM Mg^2+^. After identifying conditions to minimize the change of the g(-90 mV)/g(MIC) ratio during activation of NMDA receptors, including recording in the presence of Ba^2+ ^and using low concentrations of agonist, it was possible to repeatedly measure the g(-90 mV)/g(MIC) throughout the duration of NMDA application with minimal variation in the ratio in many of the neurons tested. The ratio values observed, particularly in NK1R+ neurons, indicated that functional receptors composed of NR2A/B and NR2C/D subunits are present. The reversible shift of the ratios to larger values in the presence of the NR2A/B antagonist, EAB318, and to smaller values in the presence of the NR2C/D preferring antagonist, UBP141, confirm this interpretation. In addition, the action of these drugs in shifting the measured g(-90 mV)/g(MIC) ratio in the predicted direction strongly supports the use of this ratio assay to identify natively expressed NR2 subunits.

### NR2A/B and NR2C/D subunits expressed by subpopulations of lamina I neurons

While published evidence suggests expression of both NR2A/B and NR2C/D subunit types in the dorsal horn generally, our data, collected on subpopulations of lamina I neurons, show cell specific differences. Previous reports indicate that NMDA receptors with NR2A and NR2B subunits are expressed in superficial dorsal horn based on *in situ *hybridization [31-33], single cell PCR [34] and immunostaining [14,35,36] studies. Our observations show strong evidence of NR2A and/or NR2B expression in both NK1R+ and NK1R- lamina I neurons. Earlier studies suggest that NR2D mRNA is expressed by many and NR2C mRNA by few dorsal horn neurons [34]. In addition, more NR2D mRNA is expressed in adult dorsal horn and embryonic spinal cord than NR2C mRNA [5,37,38]. Further support for the presence of NR2D is that NMDA receptors with NR2D-like single channel conductance have been reported for lamina II neurons in rat dorsal horn [39,40]. Based on our experiments, we have found that NK1R+ neurons express NR2C/D subunits more strongly than the NK1R- neurons. While it remains uncertain which NMDA receptors with low Mg^2+ ^sensitivity are expressed by these lamina I neurons, NR2D is the best candidate.

NK1R+ lamina I neurons represent a comparatively uniform population of neurons that are predominantly projection neurons [21]. The NK1R- neuron population is heterogeneous, including inhibitory and excitatory interneurons as well as a small population of NK1R- projection neurons [41]. Within the NK1R- population of neurons, some of the variability of NR2 subunit identity may represent different receptor configurations on different subpopulations of dorsal horn neurons.

At the whole cell level, particularly for NK1R+ neurons, we have evidence that NMDA receptors with NR2C/D subunits are present. NMDA receptors with these less Mg^2+ ^sensitive NR2 subunits could be expressed at synapses, extrasynaptically or both. Momiyama (2000) has suggested an extrasynaptic localization of NR2D containing NMDA receptors by lamina II neurons in the dorsal horn. Because of their higher binding affinity with glutamate, these extrasynaptic receptors may be more sensitive to ambient glutamate levels in the extracellular space that could accumulate due to glial release [42,43], spill over associated with high amounts of activity, and to injury [44]. Activation of these receptors would be expected to have a potent impact on neuronal cell function due to their lowered Mg^2+ ^sensitivity, prolonged time over which they open following glutamate binding, and lack of desensitization [5,6,45].

### Other factors that could influence NMDA receptor conductance ratio

One concern with our approach to NR2 subunit identification is the possibility that changes in membrane currents secondary to NMDA receptor activation will alter the g(-90 mV)/g(MIC). It is because of this concern that we have recorded in low Ca^2+ ^solution with added Ba^2+ ^and limited our analysis to those neurons showing no change in g(-90 mV)/g(MIC) while NMDA washes on and off the spinal cord slices. Even more importantly, we have used pharmacological tools as an independent test of subunit composition under these carefully controlled drug application conditions. In some of the neurons excluded from these studies, NMDA-induced currents showed strongly increased g(-90 mV)/g(MIC) ratios during wash-out of NMDA (data not shown). The underlying mechanism for this is not clear. For the data that met the criteria for our study, we have confirmed identification of subunit composition by the use of NMDA receptor specific compounds to alter conductance ratio in predictable ways. The opposing effects of EAB318 and UBP141 on g(-90 mV)/g(MIC) supports our interpretation of conductance ratio in terms of subunit composition.

### Significance

We have taken advantage of the Mg^2+ ^sensitivity of NMDA receptors to identify NMDA receptors of different NR2 subunits in identified subpopulations of lamina I neuorns and confirmed this with pharmacology. We show that individual neurons express NMDA receptors with different NR2 subunits at different ratios. When comparing identified populations of lamina I neurons, NK1R+ neurons express a higher mean ratio of NR2C/D type NMDA receptors compared with NK1R- neurons. NR2D has been suggested to have a role in the development of allodynia or hyperalgesia in several different pain models [12] and lamina I, NK1R+ neurons are importantly involved in the expression of allodynia [46]. In this context, it is possible that these receptors may contribute to development of NR2D-dependent allodynia.

## Competing interests

The authors declare that they have no competing interests.

## Authors' contributions

CKT, EJK and ABM conceived and designed the experiments. CKT and ABM analyzed the data and wrote the manuscript. CKT carried out the experiments. EJK provided the essential compounds UBP141 and EAB318. All authors read and approved the final manuscript.
